# Environmental DNA metabarcoding for fish community analysis in backwater lakes: A comparison of capture methods

**DOI:** 10.1371/journal.pone.0210357

**Published:** 2019-01-31

**Authors:** Kazuya Fujii, Hideyuki Doi, Shunsuke Matsuoka, Mariko Nagano, Hirotoshi Sato, Hiroki Yamanaka

**Affiliations:** 1 Fukuda Hydrologic Center, Sapporo, Hokkaido, Japan; 2 Research Faculty of Agriculture, Hokkaido University, Sapporo, Hokkaido, Japan; 3 Graduate School of Simulation Studies, University of Hyogo, Minatojima-minamimachi, Kobe, Japan; 4 Faculty of Science and Technology, Ryukoku University, Shiga Japan; CSIR-National Institute of Oceanography, INDIA

## Abstract

The use of environmental DNA (eDNA) methods for community analysis has recently been developed. High-throughput parallel DNA sequencing (HTS), called eDNA metabarcoding, has been increasingly used in eDNA studies to examine multiple species. However, eDNA metabarcoding methodology requires validation based on traditional methods in all natural ecosystems before a reliable method can be established. To date, relatively few studies have performed eDNA metabarcoding of fishes in aquatic environments where fish communities were intensively surveyed using multiple traditional methods. Here, we have compared fish communities’ data from eDNA metabarcoding with seven conventional multiple capture methods in 31 backwater lakes in Hokkaido, Japan. We found that capture and field surveys of fishes were often interrupted by macrophytes and muddy sediments in the 31 lakes. We sampled 1 L of the surface water and analyzed eDNA using HTS. We also surveyed the fish communities using seven different capture methods, including various types of nets and electrofishing. At some sites, we could not detect any eDNA, presumably because of the polymerase chain reaction (PCR) inhibition. We also detected the marine fish species as sewage-derived eDNA. Comparisons of eDNA metabarcoding and capture methods showed that the detected fish communities were similar between the two methods, with an overlap of 70%. Thus, our study suggests that to detect fish communities in backwater lakes, the performance of eDNA metabarcoding with the use of 1 L surface water sampling is similar to that of capturing methods. Therefore, eDNA metabarcoding can be used for fish community analysis but environmental factors that can cause PCR inhibition, should be considered in eDNA applications.

## Introduction

Ecological community evaluation is a critical step because it provides the basic information needed for biological conservation, for example the composition of fish communities in freshwater systems [1]. Previously, fish capture methods such as the use of nets and other types of fishing gear/equipment have been used for community evaluation. However, each capture method has been shown to incompletely detect fish species in a community because of differences in the traits and habitats of fish. Thus, evaluation of fish communities should be completed using several capture methods [2]. Some capture methods are difficult to employ in some ecosystems. For example, examining fish species in backwater environments is difficult because of limited access to pelagic areas, which is further complicated by the presence of macrophytes and muddy sediments. Using environmental DNA (eDNA) methods, especially DNA metabarcoding, may be a valuable new survey method for backwater habitats.

eDNA obtained directly from environmental samples can be used to evaluate species distributions. These methods have recently been developed and are considered to be useful techniques [3–8]. For example, in the past decade, many studies detected fish species [9, 10] and aquatic organisms [11–17] using eDNA. Recently, high-throughput parallel DNA sequencing (HTS) has been applied in eDNA studies to examine community composition from eDNA samples [3, 5, 18–24]. This eDNA technique with HTS sequencing and DNA-based species identification is called eDNA metabarcoding and is considered to be a useful method for assessing aquatic communities [19, 20].

eDNA metabarcoding has recently been applied in fish community surveys. For example, a universal polymerase chain reaction (PCR) primer for fish species, called MiFish (MiFish-U/E) was developed, whereby a hypervariable region of the mitochondrial 12S rRNA gene can be amplified [25]. The versatility of these PCR primers using eDNA from four aquaria was tested with known species composition and natural seawater [25]. These authors successfully detected eDNA from 232 fish species across 70 families and 152 genera in the aquaria and in the field, with a higher detection rate for species (>93%) in the aquaria. Moreover, using the MiFish primers and HTS, an investigation of marine fish communities in Maizuru Bay, Japan, detected a total of 128 fish species in the water samples [26, 27]. These studies indicate the great potential of eDNA metabarcoding as a useful tool for biodiversity assessment.

eDNA metabarcoding has been applied in fish biodiversity surveys, but testing and comparing its usefulness with traditional methods is necessary for the development of this technique as a conservation tool [28]. The performance of eDNA metabarcoding has been tested in some studies and compared with that of capture methods [29, 30] or underwater visual consensus [26, 27], and it was found to have similar or higher performance than that of traditional methods. Comparisons of species detected using eDNA with those detected using multiple capture methods, which are generally used to investigate fish communities in aquatic habitats, are limited except for a study in a marine bay [26, 27]. Moreover, eDNA metabarcoding studies have primarily been conducted in marine [25], lake [31, 32], pond [33], and river ecosystems [34–37] but not in backwater ecosystems where there are many rare and endangered fish species [38]. Therefore, a comparison of the performance of eDNA metabarcoding in assessing fish communities with that using traditional methods is necessary.

The objective of this study was to evaluate the performance of eDNA metabarcoding using HTS for fish communities in backwater lakes that are inhabited by rare and endangered fish species, and compare the ability of this methodology to detect species in a community with that of multiple capture methods, such as net sampling and electro-fishing. We conducted field surveys and sampling of eDNA in backwater lakes, including oxbow lakes that are isolated from rivers and backwater lakes, and thus, are postulated as the backwater of the rivers occurring from natural embankments [39]. We also tested PCR inhibition effect on the metabarcoding analysis in the backwater lakes. Estimations of species distributions in these lakes using eDNA have not been widely attempted, and a direct capture of fish is difficult because of high densities of macrophytes on the surface.

## Materials and methods

### Ethics statement

This project, including field survey and sampling for fish, was conducted in accordance with the Act on Welfare and Management of Animals, and Bylaw on Welfare and Management of Animals in Hokkaido, and the Regulations for Animal Experiments of the University of Hyogo. Permission for fish samplings in the backwaters and rivers in this study was granted by the Hokkaido government, Japan. An ethics statement is not required for this project.

### Study sites

**Fig 1:**
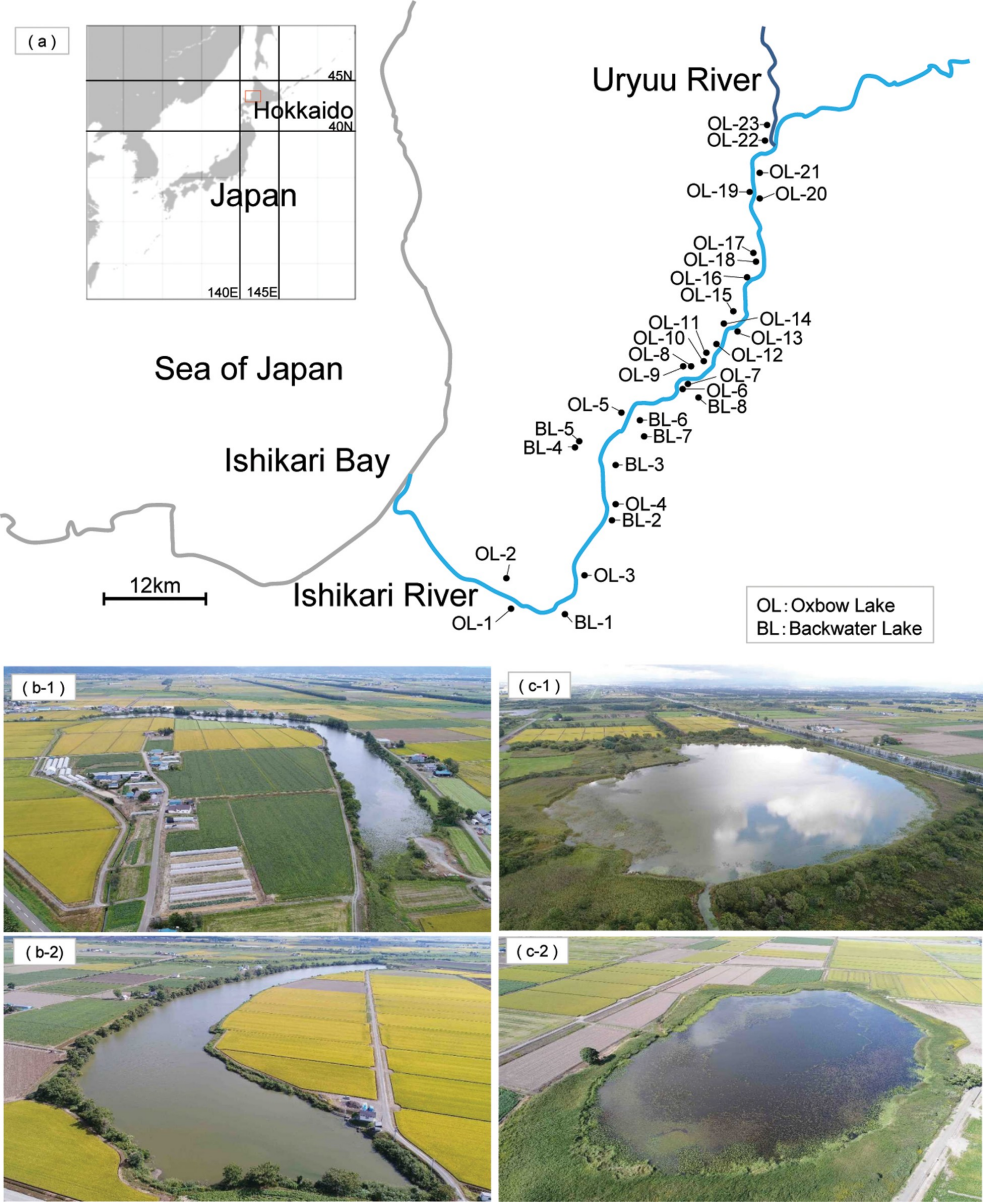
Map. a) sampling sites, b-1, 2) aerial photograph of oxbow lakes (b-1; OL-8, b-2; OL-7), and c-1, 2) aerial photograph of backwater lakes (c-1; BL-1, c-2; BL-7).

We conducted a field survey and sampling in 31 backwater lakes in Hokkaido, Japan (Fig 1A). The study lakes were classified as oxbow (OL-, Fig 1B) and backwater lakes (BL-, Fig 1C). Most of the study lakes were interconnected by canals and rivers. These lakes host many rare and endangered fish species such as *Lefua nikkonis* and *Rhynchocypris percnurus sachalinensis* [38].

### Traditional capture methods to estimate fish community composition

Traditional surveys for fish in the 31 lakes in our study were conducted, with moderate water levels, by the Hokkaido Regional Development Bureau between August 2 and September 13, 2016. The captures were conducted at shore sites. For captures, multiple fishing equipment were used including minnow traps, long-line fishing, gill nets, cast nets, dip nets, drag nets, and electro-fishing (Table 1). For minnow traps and long-line fishing, we used Pacific saury as bait. These traditional methods have been conducted for fish community surveys by the government such as the Ministry of Land, Infrastructure and Transport, Japan, and researchers in the habitats. The multiple fishing equipment may catch most of the fish species occupying the habitats. All capture methods were used for all sites and capture efforts were equal across all study lakes. Fish captured using fishing equipment were pooled and their abundances were calculated. Species were identified using pictorial keys [40]. Because the individuals were juvenile and ammocoetes, some species were difficult to identify to species level and were included as a genus.

**Table 1:** Capture methods employed in this study, including the fishing equipment size and effort.

Fishing methods	Gears and sizes	Fishing Effort
Long-line fishing	Line-length 15 m Number of baited Hooks 10/Line	2 lines/site 1 night
Minnow trap	L60 cm × W45 cm × H20 cm mesh size 25 mm	10 pcs/site 1 night
Gill net	W10 m × H3 m Mesh size 85 mm and 106 mm	1 gear/site 1 night for each size
Cast net	Mesh size 12 mm and 18 mm	10~20 cast/site
Dip net	Bow 80 cm × 60 cm Net-depth 1.0 m Mesh size 5 mm	30 min × 1 person/site
Dragnet	Net-height 2.0 m Net-length 18 m Collect parts mesh size 15 mm Capture parts mesh size 5 mm	2 times/site
Electro-fisher	Type LR-12B (Smith–Root, Inc., Vancouver, WA, USA)	60 min/site

### Water sampling and filtering

Using the aforementioned methods, we collected 1 L of surface water in bleached bottles from the shores of each lake where fish had previously been caught. We conducted water sampling, with moderate water levels in the lakes, from October 31 to November 4, 2016. Water samples were vacuum-filtered on-site onto 47 mm GF/F glass filters (pore size: 0.7 μm) (GE Healthcare, Little Chalfont, UK). During transport, the filters were stored in a cooler with ice packs. All filters were stored at -20°C within 12 h after filtration. A liter of Milli-Q water was used as the equipment control to monitor contamination during filtering in each site and during subsequent DNA extraction. The sampling bottles and filtering equipment (i.e., filter funnels and measuring cups) were cleaned using 10% commercial bleach (approximately 0.6% hypochlorous acid) and washed using DNA-free distilled water.

### eDNA extraction from filter samples

To extract DNA from the filters, we followed previously described methods [22] using DNeasy Blood and Tissue Kit for DNA purification (Qiagen, Hilden, Germany) and Salivatte (Sarstedt, Numbrecht, Germany). The filter was placed in a salivated tube, and Buffer AL (400 μL) and proteinase K (20 μL) were added to the solution. The column was placed in a 56°C dry-oven for 30 min. After incubation, the salivated tube was centrifuged at 6,000 × *g* for 1 min to collect the DNA. To increase DNA yield from the filter, 220 μL of TE buffer was added to the filter and again centrifuged at 6,000 × *g* for 1 min. The collected DNA was purified using the DNeasy Blood and Tissue Kit following the manufacturer’s protocol. The extracted DNA samples (100 μL) were stored at –20°C until PCR assay.

### Library preparation and MiSeq sequencing

A two-step PCR-procedure was used for library preparation of Illumina MiSeq sequencing. As the first step, a fragment of the mitochondrial 12S rRNA gene was amplified using the MiFish-U-F and MiFish-U-R primers [25] which were designed to contain Illumina sequencing primer regions and 6-mer Ns (forward: 5′-ACACTCTTTCCCTACACGACGCTCTTCCGATCTNNNNNN GTCGGTAAAACTCGTGCCAGC-3′, reverse: 5′-GTGACTGGAGTTCAGACGTGTGCTCTTCCGATCTNNNNNN CATAGTGGGGTATCTAATCCCAGTTTG-3′). The italicized and normal letters represent MiSeq sequencing primers and MiFish primers, respectively, and the six random bases (N) were used to enhance cluster separation on the flow cells during initial base call calibrations on MiSeq. We used a KOD FX Neo polymerase (Toyobo, Osaka, Japan) for the first PCR to facilitate amplifications of DNA from crude extracts. The first PCR was performed with a 12 μL reaction volume containing 1× PCR Buffer for KOD FX Neo, 0.4 mM dNTP mix, 0.24 U KOD FX Neo polymerase, 0.3 μM of each primer, and 2 μL template [41]. The thermal cycles of this step were as follows: initial denaturation at 94°C for 2 min, followed by 35 cycles of denaturation at 98°C for 10 s, annealing at 65°C for 30 s, and elongation at 68°C for 30 s, followed by final elongation at 68°C for 5 min. The first PCRs were performed using eight replicates to mitigate false negatives (PCR dropouts). Thereafter, individual 1^st^ PCR replicates were pooled. The 30 μL of each PCR product was purified using AMPure XP (Beckman Coulter, Brea CA, USA) and eluted with 30 μL of sterilized water. The purified first PCR products were used as templates for the second PCR. The Illumina sequencing adaptors and the 8 bp identifier indices were added to the subsequent PCR process using a forward and reverse fusion primer: 5´-AATGATACGGCGACCACCGAGATCTACAXXXXXXXXACACTCTTTCCCTACACGACGCTCTTCCGATCT-3´ (forward) and 5´-CAAGCAGAAGACGGCATACGAGATXXXXXXXXGTGACTGGAGTTCAGACGTGTGCTCTTCCGATCT-3´ (reverse). The italic and normal letters represent MiSeq P5/P7 adapter and sequencing primers, respectively. The 8X bases represent dual-index sequences inserted to identify different samples. The second PCR was conducted with 12 cycles of a 12 μL reaction volume containing 1× KAPA HiFi HotStart ReadyMix, 0.3 μM of each primer, and 1.0 μL template from the first PCR production. The thermal cycle profile after an initial 3 min denaturation at 95°C was as follows: denaturation at 98°C for 20 s, annealing, and extension combined at 72°C (shuttle PCR) for 15 s, with the final extension at the same temperature for 5 min. The second PCR products were pooled in equal volumes and purified using AMPure XP as the first PCR. The purified PCR products were loaded on a 2% E-Gel SizeSelect (Thermo Fisher Scientific, Waltham, MA, USA) and the target size of the libraries (approximately 370 bp) was collected. The library concentration and quality were estimated by a Qubit dsDNA HS assay kit and a Qubit 2.0 (Thermo Fisher Scientific). The amplicon libraries were sequenced by 2 × 250 bp paired-end sequencing on the MiSeq platform at Ryukoku University using the MiSeq v2 Reagent Kit according to the manufacturer’s instructions. Note that the sequencing run contained a total of 351 libraries including 34 of our libraries and 317 libraries from other research projects.

### Bioinformatic analysis for MiSeq sequencing

The processing formality of the MiSeq reads was evaluated by the FASTQC program (http://www.bioinformatics.babraham.ac.uk/projects/fastqc). After confirming a lack of technical errors in the MiSeq sequencing, low-quality tails were trimmed from each read using “DynamicTrim.pl” in the SOLEXAQA software package [42] with a cut-off threshold set at a Phred score of 10. The trimmed paired-end reads (reads 1 and 2) were then merged with each other. The assembled reads were further filtered by custom Perl scripts to remove reads with either ambiguous sites (Ns) or those exhibiting unusual lengths with reference to the expected size of the PCR amplicons (297 ± 25 bp). The software TagCleaner [43] was used to remove primer sequences with a maximum of three-base mismatches and transform the FASTQ format into FASTA.

The pre-processed reads with an identical sequence (i.e., 100% sequence similarity) were assembled using UCLUST [44]. The number of identical reads was added to the header line of the FASTA formatted data file. The sequences represented by more than or equal to 10 identical reads were subjected to the downstream analyses.

**Fig 2:**
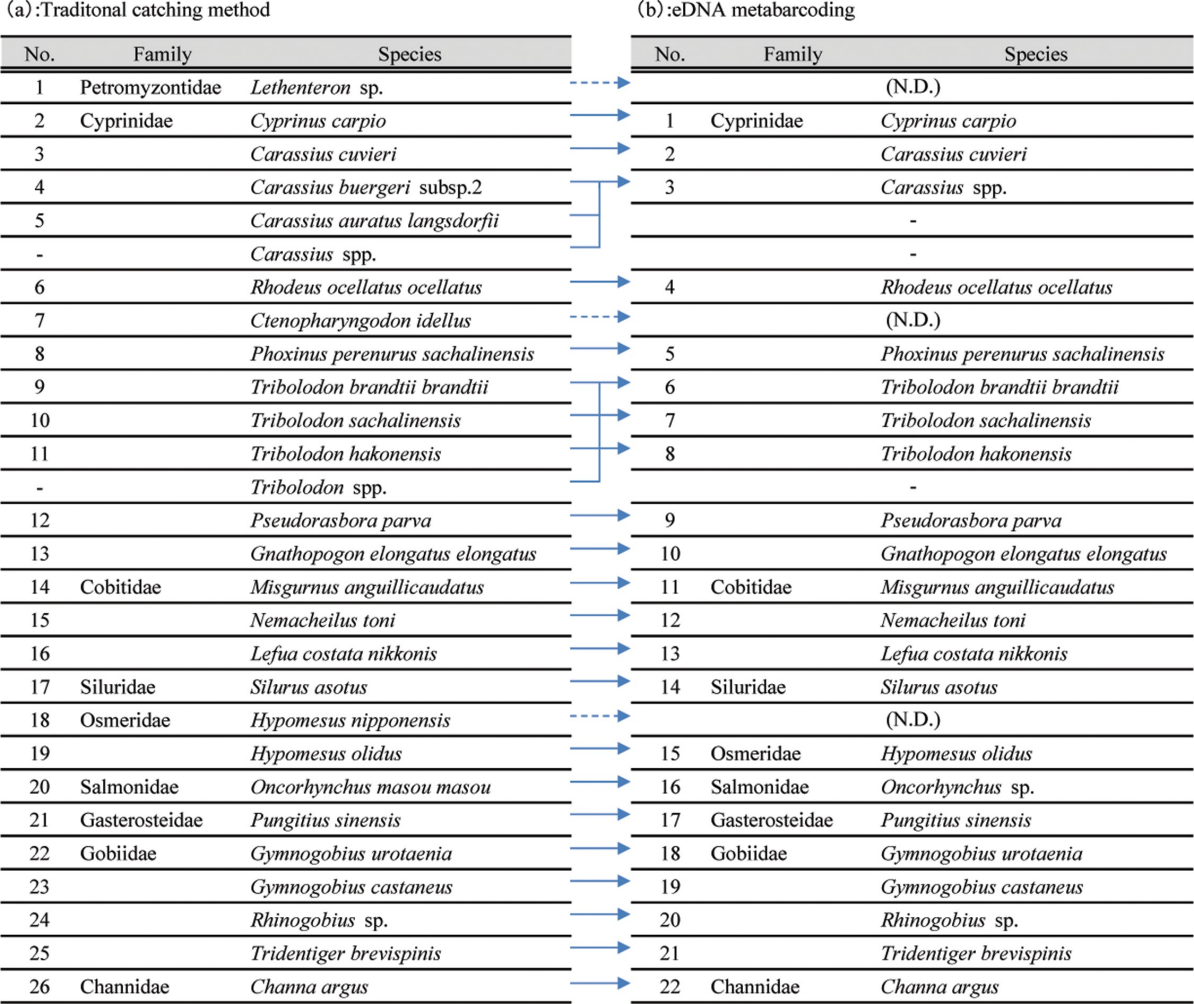
Species lists determined from captures (a) and eDNA metabarcoding (b).* N.D. means that the species was not detected.

The processed reads were subjected to local BLASTN searches on the comprehensive reference database of fish species that were previously established [25]. The top BLAST hit with a sequence identity of ≥97% and the E-value threshold of 10^−5^ was applied to species detection of each sequence, but the species were mostly identified with ≥99% match (Fig 2). From the BLAST results, we identified the species using methods previously described [41]. We also calculated the rate of shared sites by the number of sites with species detected by metabarcoding per number of sites with species detected by metabarcoding and traditional methods.

### PCR inhibition test

To test for inhibition in the eDNA samples, 1 μL of the plasmid including the cytochrome b gene from *Trachurus japonicus* (1.5 × 10^2^ copies), a marine fish, which was not found at the sites and detected by the eDNA metabarcoding, was added to the PCR template. This contained 900 nM of each primer and 125 nM of TaqMan probe in a 1× PCR master mix (KOD FX Neo) and 2 μL of the DNA solution. The total volume of each reaction mixture was 10 μL. The real-time PCR was performed by using quantitative real-time PCR (PikoReal real-time PCR, Thermo Fisher Scientific). The PCR (three replicates) was performed as follows: 2 min at 50°C, 10 min at 95°C, and 55 cycles of 15 s at 95°C, and 60 s at 60°C. The non-template control (NTC) was performed in three replicates per PCR. The results of the PCR were analyzed using PikoReal software v. 2.2.248.601 (Thermo Fisher Scientific). The primer and probe set used was that previously reported [26]: forward primer: 5′-CAGATATCGCAACCGCCTTT-3′; reverse primer: 5′-CCGATGTGAAGGTAAATGCAA A-3′; probe: 5′-FAM-TATGCACGCCAACGGCGC CT-TAMRA-3′. The presence of PCR inhibitors was evaluated ΔCt (Ct_positive control_−Ct_sample_). ΔCt of ≥3 cycles and is usually considered to be evidence of inhibition [45].

### Statistical analyses

To evaluate the overlapping of the species lists between the captured fish and eDNA metabarcoding, we calculated the detection rate for eDNA metabarcoding. The equation was (the number of species found by both the capture and eDNA methods/number of all captured species) × 100. We conducted the community analysis for both the eDNA metabarcoding and catching data. We used the presence/absence data of both datasets and calculated the Jaccard similarity index and ordinated using non-metric multidimensional scaling (NMDS) with 100 iterations. We also performed an analysis of similarities (ANOSIM) for the Jaccard similarity index to test whether there was a statistically significant difference between two or more groups of sampling units. We tested the differences in communities between the eDNA metabarcoding and catching data (permutation = 999, α = 0.05). All statistical analyses were conducted in the “vegan” package of R v. 3.3.2 (R Core Team 2016) [46].

## Results

### Traditional catching methods to estimate fish community

Using multiple capture methods, we found 26 taxa in nine families (Fig 2A). Cyprinidae was the most abundant family (12 species).

### MiSeq sequencing

A MiSeq paired-end sequencing for the 34 PCR libraries (including 31 samples and three negative controls) yielded a total of 641.056 reads. We detected 24 taxa in eight families after following the pipeline procedure for eDNA metabarcoding, representing 85% of taxa that we found by the traditional methods (Fig 2B). We identified *Carassius buergeri* and *C*. *auratus langsdorfii* as *Carassius* spp. because of higher DNA-sequence similarities (<2 bp differences). We also detected two invaive species in this region, namely, *Opsariichthys uncirostris* and *Zacco platypus*. Using eDNA metabarcoding, we could not find three fish species that were detected by capture methods (i.e., *Lethenteron* sp., *Ctenopharyngodon idella*, and *Hypomesus nipponensis* at any site (Table 2).

**Table 2:** Species determined from capture approaches (○), eDNA metabarcoding (△), and both methods (●).

Species	OL																							BL							
	1	2	3	4	5	6	7	8	9	10	11	12	13	14	15	16	17	18	19	20	21	22	23	1	2	3	4	5	6	7	8
*Lethenteron* sp.							○																								
*Cyprinus carpio*	●	●	●	●	●	●	○	●	●	●	●	●	△	○		●	△	○	●	●	○	○	●				△	○	△		
*Carassius cuvieri*	●	●	●	●	●	●	●	●	●	●	●	●	△	○	○	●	●	○	○	●	△		●				●	○	●	△	○
*Carassius* spp.	●	●	●	●	●	●	●	●	△	●	●	●	●	○	○	○	△	●	○	●	●	●	○	○	●	○	●	○	●	●	●
(*C*.*buergeri* subsp.2)																															
(*C*. *auratus langsdorfii*)																															
(*C*.spp.)																															
*Rhodeus ocellatus ocellatus*	●	●	●	●	●	●	●	●	●	●	●	●	△	○	○	○	●	●	○	●	○	○	○	○	○			○	●		●
*Ctenopharyngodon idellus*														○																	
*Phoxinus perenurus sachalinensis*										△		△	△				△		○	△	○	●	○	○	○	●	○	○		●	●
*Tribolodon brandtii brandtii*	△	△		△	△	△	△	△	△	△	●	△	△							△								○	△		
*Tribolodon sachalinensis*	●	△		△	●	●		●	●	●	●	●	●	○	○	●		●	△	●	△	●	●		○		△	○			
*Tribolodon hakonensis*	△	△	○	△	△	●	△	△	△	●	△	△	△			△	△		○	●	●	△									
(*Tribolodon* spp.)		○	○	○	○	○	○	○	○		○	○	○	○	○	○		○	○	○	○	○	○		○				○		
*Pseudorasbora parva*	●	●	●	●	●	●	●	●	●	△	●	●	●	○	○	●	●	●	●	●	○	○	○	○	○		●	○	●	●	○
*Gnathopogon elongatus elongatus*		●	●	●	△	●		●	●	●	●	●	●	○		●	△	●	●	●	○							○	○	△	
*Misgurnus anguillicaudatus*	△	△	△	●	△	●	●	●	●	●	●	△	●	○	○	●	○	●	●	●	○	●	●						△		○
*Nemacheilus toni*		△			●			●		△	●	△	●			●		△	○	○	●	●	△								
*Lefua costata nikkonis*	△				△							△							○		○	●	●							△	○
*Silurus asotus*		●	●		○	○	○	○	●	○	○	○	○	○		○	○						●								
*Hypomesus nipponensis*											○																				
*Hypomesus olidus*		○	○		●	●	○	●	●	○	○			○	○	○	●	△		○			○	○			●	○	●		○
*Oncorhynchus* sp.	△				△											○															
(*Oncorhynchus masou masou*)																															
*Pungitius sinensis*	△	△			△			●	△	●	●	●	△		○	○	●	○	○		○	●	●	○	●	○	●	○		●	●
*Gymnogobius urotaenia*	●	△		△		●			●																						
*Gymnogobius castaneus*		○	●	●	●	●	●	○	●	●	●	●	●	○	○	○	●	○		●	●		○	○	○		●	○	●	△	
*Rhinogobius* sp.		△	●	○	○	●	●	○	●	●	○	○	●	○		○	●	○	○	●	●		○				●	○			
*Tridentiger brevispinis*					△	●																							△		
*Channa argus*	●			●	●		●		●	○		●			○	●	●	○	○												

In OL-14, OL-15, BL-1, and BL-5, we could not detect any fish species, probably because the PCR amplification almost failed due to PCR inhibitors. In fact, PCR inhibitor tests using real-time PCR showed remarkable inhibition (∆Ct > 3) in the samples compared with other sites and that showed modulate inhibition (∆Ct = 1.87) at BL-1 (Table 3). We also detected marine species using eDNA metabarcoding (Table 4). The number of reads for each of these species was one or two orders of magnitude less than that for expected species.

**Table 3:** The mean ΔCt for PCR inhibitor tests for the extracted samples.

Site	ΔCt	eDNA metabarcoding	Site	ΔCt	eDNA metabarcoding
OL-1	-0.26	Detected	OL-17	0.22	Detected
OL-2	-0.29	Detected	OL-18	-0.11	Detected
OL-3	-0.19	Detected	OL-19	0.25	Detected
OL-4	-0.44	Detected	OL-20	-0.15	Detected
OL-5	-0.21	Detected	OL-21	0.31	Detected
OL-6	-0.29	Detected	OL-22	-0.17	Detected
OL-7	-0.21	Detected	OL-23	0.25	Detected
OL-8	-0.27	Detected			
OL-9	-0.10	Detected	BL-1	1.87	Not Detected
OL-10	0.06	Detected	BL-2	0.16	Detected
OL-11	0.17	Detected	BL-3	-0.20	Detected
OL-12	-0.04	Detected	BL-4	0.25	Detected
OL-13	0.11	Detected	BL-5	4.57	Not Detected
OL-14	12.48	Not Detected	BL-6	-0.37	Detected
OL-15	6.54	Not Detected	BL-7	-0.50	Detected
OL-16	0.07	Detected	BL-8	-0.62	Detected

**Table 4:** Marine fish species detected using eDNA metabarcoding and the total reads by high-throughput parallel DNA sequencing (HTS).

No.	Family	Species	Common name	eDNA Detective Site	Total reads	BLAST Identity (%)
1	Clupeidae	*Sardinops melanostictus*	Japanese sardine	OL-1	15	100
2	Clupeidae	*Clupea pallasii*	Pacific herring	OL-1	23	100
3	Gadidae	*Gadus chalcogrammus*	Alaska pollock	OL-1	27	100
4		*Gadus morhua*	Atlantic cod	OL-1,OL-21,OL-22	148	100
5		*Gadus macrocephalus*	Pacific cod	OL-1	13	100
6	Scomberesocidae	*Cololabis saira*	Pacific saury	OL-1,OL-22	60	100
7	Sebastidae	*Sebastes trivittatus*	-	OL-1	134	100
8		*Sebastes ruberrimus*	Yelloweye rockfish	OL-1	21	100
9	Hexagrammidae	*Pleurogrammus azonus*	Okhotsk atka mackerel	OL-1	32	100
10	Carangidae	*Seriola quinqueradiata*	Japanese amberjack	OL-1	199	99.4
11	Sparidae	*Pagrus major*	Japanese seabream	OL-1	70	100
12	Scombridae	*Scomber australasicus*	Blue mackerel	OL-1	17	100
13	Pleuronectidae	*Pleuronectes schrenki*	Flounder	OL-1	19	100
14		*Pseudopleuronectes americanus*	Winter flounder	OL-1	233	100
15		*Microstomus achne*	Slime flounder	OL-1	11	100

### Comparisons of traditional capture and eDNA metabarcoding data

**Fig 3:**
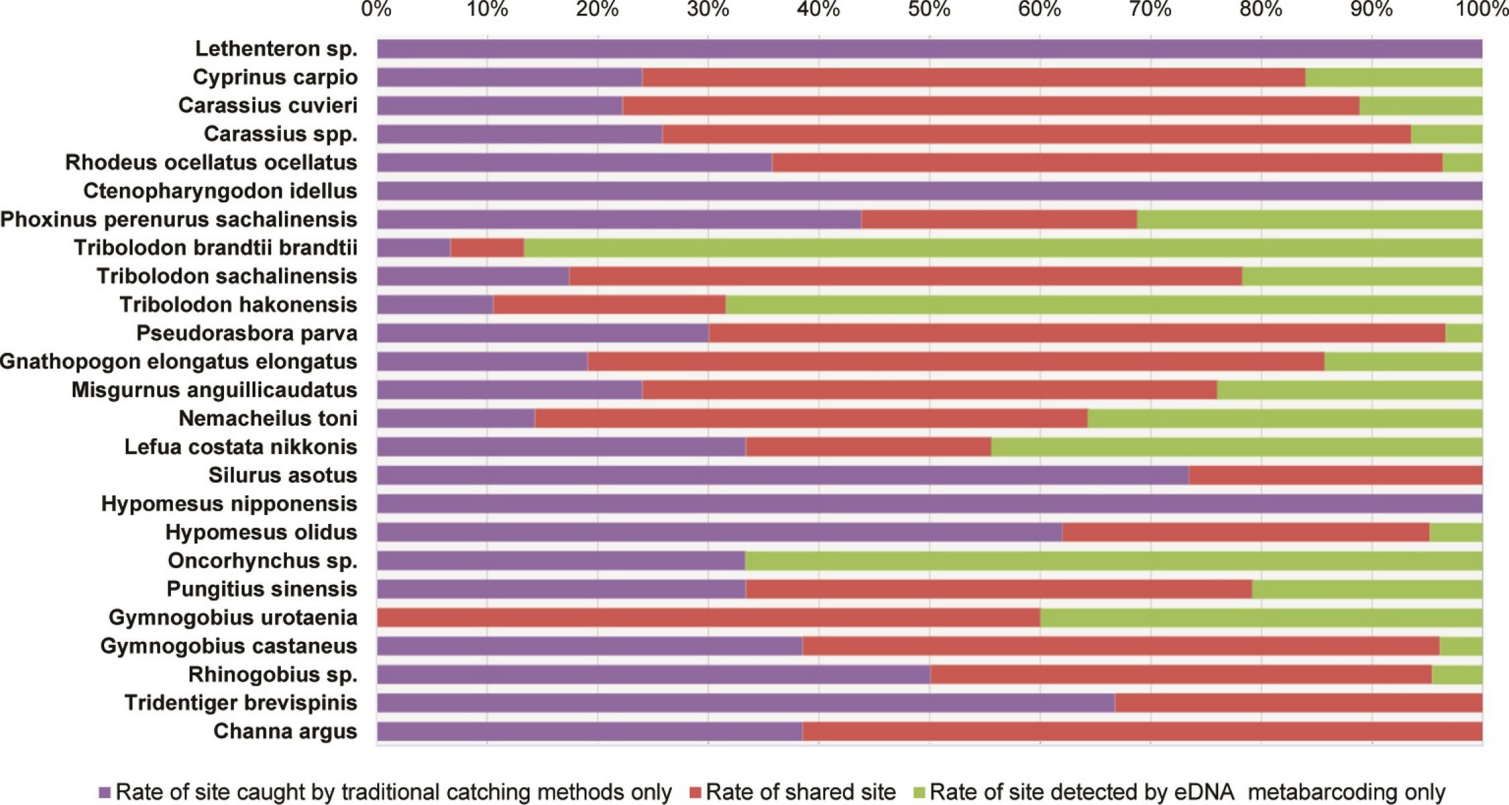
Detections of species in study lakes detected by multiple capture and eDNA metabarcoding methods.

**Fig 4:**
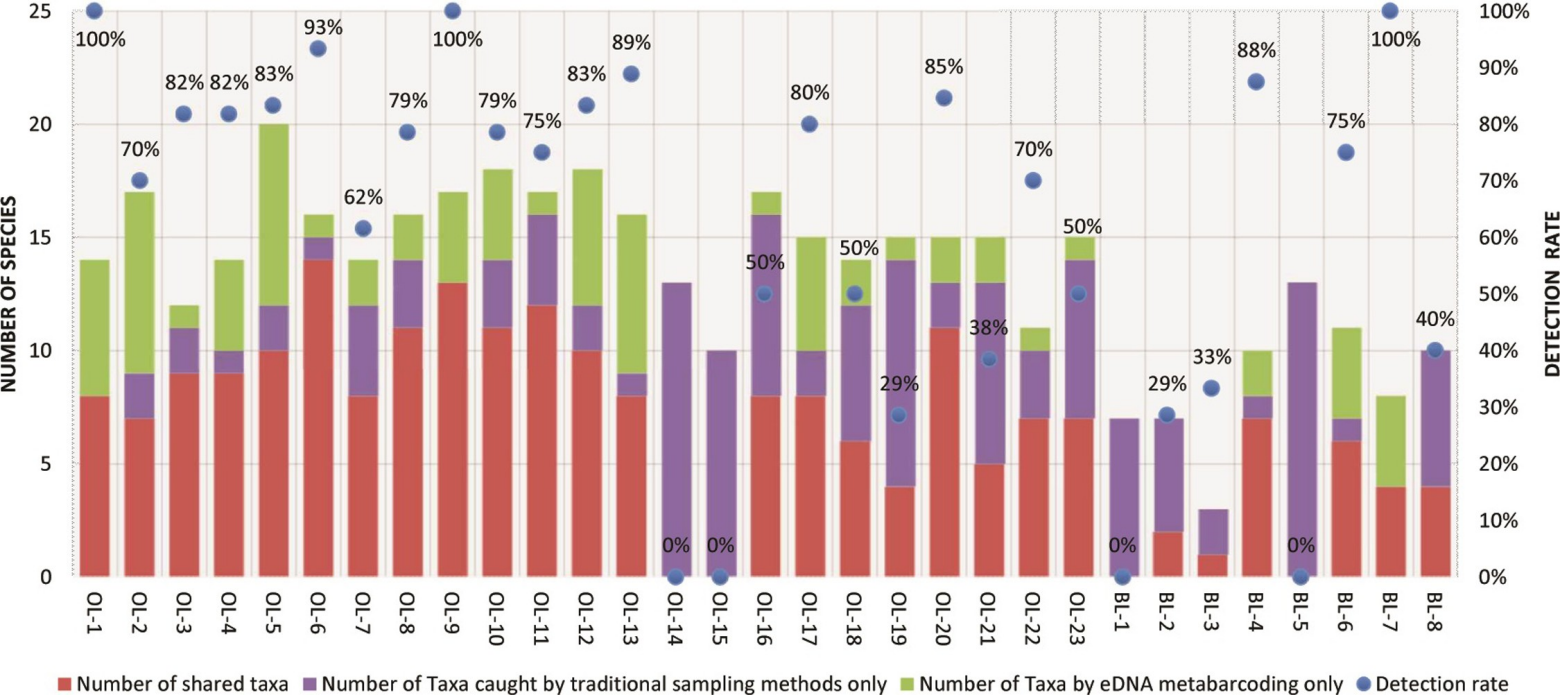
Species number and detection rate compared between eDNA metabarcoding and capture methods.

To compare between traditional capture methods and eDNA metabarcoding data, the taxonomic levels in the species list from traditional captures were adjusted to the lists from eDNA metabarcoding (Fig 2). Rates of shared sites were higher than 50% in 12 out of the 25 taxa (Fig 3 and Table 5). Using detection rates from eDNA metabarcoding, we compared detection among the sites (Fig 4). The detection rates were >50% in 22 out of 31 sites, including rates that were 100% at three sites (Fig 4). After the exclusion of the four sites where there were no eDNA detections (OL-14, OL-15, BL-1, and BL-5), the mean detection rate was 70.0%. However, the rate varied among the sites.

**Table 5:** The species list with the number of positive sites, shared sites for multiple capture methods and eDNA metabarcoding with total reads of high-throughput parallel DNA sequencing (HTS), and the number of captured individuals.

Capture No.	eDNA detection No.	Family	Species		Number of positive sites			Number of shared site	Number of eDNA reads	Number of captures	BLAST Identitiy of eDNA reads
			eDNA method	Traditonal method		eDNA method	Traditonal method				
1	-	Petromyzontidae	*-*	*Lethenteron* sp.	1	0	1	0	0	1 (by Dip net)	-
2	1	Cyprinidae	*Cyprinus carpio*	*C*. *carpio*	25	19	21	15	19,621	92	100
3	2		*Carassius cuvieri*	*C*. *cuvieri*	27	21	24	18	90,484	304	100
4–5	3		*Carassius* sp.	*C*.*buergeri* subsp.2 *C*. *auratus langsdorfiiC*.sp.	31	23	29	21	91,034	1,219	100
6	4		*Rhodeus ocellatus ocellatus*	*R*. *ocellatus ocellatus*	28	18	27	17	26,521	1,106	99.7
7	-		*Ctenopharyngodon idellus*	*C*. *idellus*	1	0	1	0	0	1 (by Gill net)	-
8	5		*Phoxinus perenurus sachalinensis*	*P*. *perenurus sachalinensis*	16	9	11	4	2,038	366	98.9
9	6		*Tribolodon brandtii brandtii*	*T*. *brandtii brandtii*	15	14	2	1	25,675	4	100
10	7		*Tribolodon sachalinensis*	*T*. *sachalinensis*	23	19	18	14	38,905	235	100
11	8		*Tribolodon hakonensis*	*T*. *hakonensis*	19	17	6	4	24,289	25	100
-	-		*-*	*Tribolodon* sp.	24	-	24	-	-	2,203	-
12	9		*Pseudorasbora parva*	*P*. *parva*	30	21	29	20	60,005	1,473	100
13	10		*Gnathopogon elongatus elongatus*	*G*. *elongatus elongatus*	21	17	18	14	9,524	644	100
14	11	Cobitidae	*Misgurnus anguillicaudatus*	*M*. *anguillicaudatus*	25	20	19	14	25,276	242	100
15	12		*Nemacheilus toni*	*N*. *toni*	14	12	9	7	14,384	77	100
16	13		*Lefua costata nikkonis*	*L*. *costata nikkonis*	9	6	5	2	1,010	94	99.9
17	14	Siluridae	*Silurus asotus*	*S*. *asotus*	15	4	15	4	104	42	100
18	-	Osmeridae	*Hypomesus nipponensis*	*H*. *nipponensis*	1	0	1	0	0	11	-
19	15		*Hypomesus olidus*	*H*. *olidus*	21	8	20	7	3,724	2,592	100
20	16	Salmonidae	*Oncorhynchus* sp.	*Oncorhynchus masou masou*	3	2	1	0	1,243	1 (by Gill net)	100
21	17	Gasterosteidae	*Pungitius* sp.	*P*. *sinensis*	24	16	19	11	4,941	815	100
22	18	Gobiidae	*Gymnogobius urotaenia*	*G*. *urotaenia*	5	5	3	3	1,601	15	100
23	19		*Gymnogobius castaneus*	*G*. *castaneus*	26	16	25	15	34,055	1,599	99.4
24	20		*Rhinogobius* sp.	*R*. sp.	22	11	21	10	2,433	449	100
25	21		*Tridentiger brevispinis*	*T*. *brevispinis*	3	3	1	1	1,348	8	99.8
26	22	Channidae	*Channa argus*	*C*. *argus*	13	8	13	8	3,331	34	100

### Comparing the ordination of fish communities between traditional capture and eDNA metabarcoding data

**Fig 5:**
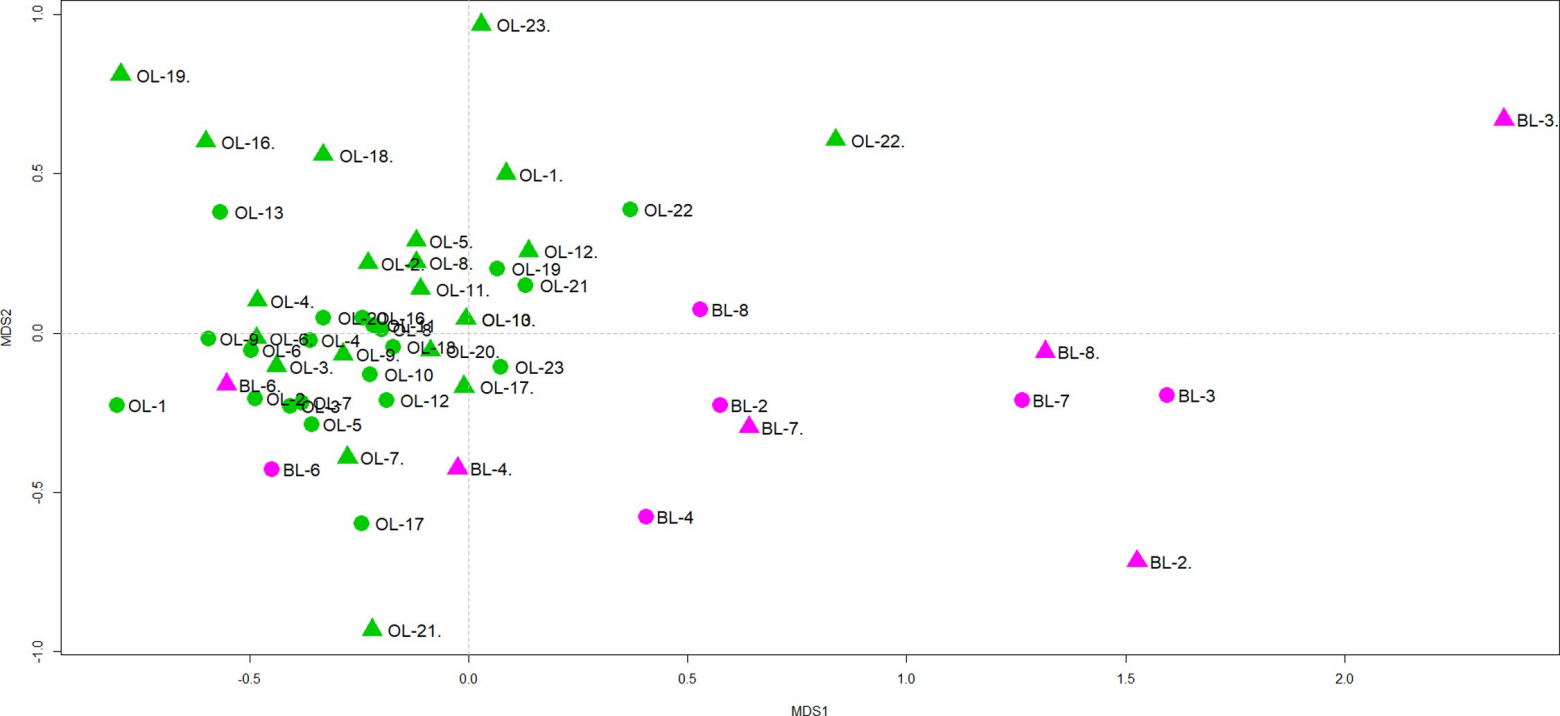
NMDS ordination for the fish community evaluated by multiple capture methods (TM, green color) and eDNA metabarcoding (MB, pink color).

The NMDS ordinations for fish community similarities were similar in structure for both traditional capture and eDNA metabarcoding data (stress value = 0.14) (Fig 5). In fact, the communities were not significantly different between traditional capture and eDNA metabarcoding using ANOSIM (R = -0.0161, *P* = 0.585). The BL sites were clearly different from the OL sites in both survey methods.

## Discussion

We found that eDNA metabarcoding using only 1 L of water sample offered high detection rate (85%) of fish taxa when multiple capture methods and eDNA metabarcoding to evaluate fish communities in backwaters were compared. Multiple fish captures using seven types of equipment required three people to be present at each site each day, whereas eDNA sampling required only one person at each site, each day and only required a few minutes to complete. Here, we showed that only 1 L of water would be sufficient to evaluate fish community composition, and we also illustrated the similarity of fish communities across our study sites using NMDS and ANOSIM results. Thus, eDNA metabarcoding can reduce the cost and effort of surveying fish communities in backwaters and other freshwater habitats.

### Comparison between the traditional capture and eDNA metabarcoding data

Some taxa that were captured using traditional methods were not detected by eDNA metabarcoding. Between 1 and 11 individuals caught using the seven capture methods were taxa that were not detected using eDNA. These were: *Lethenteron* sp., *Ctenopharyngodon idella*, and *Hypomesus nipponensis*. The low abundance of the taxa may be the result of a limited amount of eDNA in the water samples. Moreover, because *Lethenteron* sp. inhabits the sediment or sand bed of the lakes, an adequate amount of eDNA may not be available around the surface water [40], resulting in a lack of detection by eDNA metabarcoding. Such limitations of eDNA were also reported in previous real-time PCR based studies [47]. In this study, we only collected 1 L-samples from the lakes without biological replications. Increasing replications induces the detection of fish species by eDNA metabarcoding [41], thus, the limited sampling may have also caused non-detection by eDNA metabarcoding.

*Tribolodon brandtii* was caught at only two sites, but eDNA metabarcoding detected the taxa at 14 sites, probably indicating that most of the individuals identified as *Tribolodon* sp. in the field actually represent *T*. *brandtii*. In fact, individuals of *Tribolodon* genus with <10 cm body length were not identified to the taxa level in the field because of difficulties in morphological identification. For example, to identify the genus *Tribolodon* in the field, the number of predorsal scale rows must be counted and therefore, eDNA metabarcoding is advantageous where such genera are difficult to identify. An additional advantage of eDNA metabarcoding occurs in the case of some taxa for which only juveniles and ammocoetes are available and cannot be identified to species by traditional methods. The eDNA metabarcoding can identify the species regardless of the maturity stage.

We were not able to detect eDNA from four sites using metabarcoding, namely, OL-14, OL-15, BL-1, and BL-5. The results of PCR inhibitor tests showed remarkable inhibitions at these four sites (Table 3). High humic acid in the surface water of the backwater lakes in the region has been described [48]. Humic acid is a major PCR inhibitor that would reduce the number of present amplicons [49–52]. Therefore, the PCR inhibitor probably decreased the detection rate at these sites, despite the fact that we used the PCR master mix (KOD FX Neo) to amplify the DNA from samples with high PCR inhibitors, such as soil samples. Thus, we suggest the need for caution when applying eDNA metabarcoding in backwater habitats where many PCR inhibitors are present, such as in streams [53]. In this study, we extracted DNA from water samples using DNeasy blood and tissue kits. The use of another DNA extraction kit, such as the PowerSoil kit (Qiagen), might reduce the amount of humic acid extracted from samples, mitigating the negative effects of PCR inhibitors, but further study is still needed.

We also found similarities between NMDS ordinations using traditional capture methods and eDNA metabarcoding, indicating that fish community structures across sites were not significantly different between the two methods. In fact, the taxa list showed a 70% overlap. Therefore, the community analysis using eDNA can be useful for spatially broad areas with remarkably little effort.

### eDNA detections of fish species

We detected marine species using eDNA metabarcoding. In addition to marine species, *Oncorhynchus keta*, *Oncorhynchus masou masou*, *Spirinchus lanceolatus*, and *Plecoglossus altivelis*, that inhabit freshwater habitats, are often used in regional cuisine and therefore, sewage water may contain eDNA from these species. Thus, sewage water may be a source of the eDNA of unusual species in the freshwater of backwater lakes. Furthermore, marine species were exclusively detected in the OL-1 site because this site was an exception, with sewage water flowing directly into the lake. Thus, it is possible that samples collected in the lake water were contaminated by the marine fish-derived DNA from the sewage. It has been suggested that the eDNA of commonly consumed species may originate from wastewater contamination [54]. It is, therefore, necessary to consider the source of DNA release to address the false-positive detections in eDNA metabarcoding, especially if it is from sewage water.

Through eDNA metabarcoding, we also detected two domestic invasive species in OL-12 that had never been found in the region: *Opsariichthys uncirostris* (1817 reads) and *Zacco platypus* (678 reads). The species *Z*. *platypus* was found in another region of Hokkaido Island, whereas *O*. *uncirostris* had never been found on Hokkaido Island. In this study, we caught seven domestic invasive species that had previously been found in this region, including *Cyprinus carpio*, *Pseudorasbora parva*, and *Gnathopogon elongatus*. Aside from these species, *O*. *uncirostris* and *Z*. *platypus* are possibly new invasive species to the region. Although further evaluation of capture is needed, eDNA metabarcoding can identify potentially invasive species in aquatic habitats.

## Conclusion

In conclusion, eDNA metabarcoding of fish communities was performed similarly through multiple capture methods in backwater lakes. Traditional fish-capture methods, using nets or electro-fishing, require more effort and are more time-consuming in the field. Here, we showed that eDNA in 1 L water samples had a similar detectability to that of traditional methods of fish capture, suggesting the usefulness of eDNA in detecting fish community in the habitats. The eDNA metabarcoding can provide us with various information about fish communities, including species compositions of fish, emergence of new invasive species, and survival of locally extinct species. We also noted some disadvantages of the eDNA metabarcoding such as PCR inhibition for eDNA analysis and false positives of some marine species originating from wastewater contamination. Such disadvantages of eDNA methods should be considered in future applications.
